# Response to sunitinib in combination with proton beam radiation in a patient with chondrosarcoma: a case report

**DOI:** 10.1186/1752-1947-6-41

**Published:** 2012-01-30

**Authors:** Jennifer Dallas, Iman Imanirad, Rajiv Rajani, Roi Dagan, Sukanthini Subbiah, Rebecca Gaa, Wayne A Dwarica, Alison M Ivey, Robert A Zlotecki, Robert Malyapa, Danny J Indelicato, Mark T Scarborough, John D Reith, C Parker Gibbs, Long H Dang

**Affiliations:** 1 Division of Hematology and Oncology, Department of Internal Medicine, University of Florida Shands Cancer Center, University of Florida, Gainesville, FL, USA; 2 Division of Orthopedic Oncology, Department of Orthopedic Surgery, University of Florida Shands Cancer Center, University of Florida, Gainesville, FL, USA; 3 Department of Radiation Oncology, University of Florida Shands Cancer Center, University of Florida, Gainesville, FL, USA; 4 Department of Pathology, University of Florida Shands Cancer Center, University of Florida, Gainesville, FL, USA

## Abstract

**Introduction:**

Chondrosarcoma is well-known to be primarily resistant to conventional radiation and chemotherapy.

**Case presentation:**

We present the case of a 32-year-old Caucasian man with clear cell chondrosarcoma who presented with symptomatic recurrence in his pelvis and metastases to his skull and lungs. Our patient underwent systemic therapy with sunitinib and then consolidation with proton beam radiation to his symptomatic site. He achieved complete symptomatic relief with a significantly improved performance status and had an almost complete and durable metabolic response on fluorine-18-fluorodeoxyglucose positron emission tomography.

**Conclusions:**

Our findings have important clinical implications and suggest novel clinical trials for this difficult to treat disease.

## Introduction

Chondrosarcomas are malignant tumors that arise from cartilaginous tissue and comprise four major subtypes: mesenchymal, clear cell, conventional and dedifferentiated [1,2]. In about half of cases, tumors develop from large bones of the lower extremities and in one-fifth, disease is metastatic upon presentation. Except for the mesenchymal subtype, chondrosarcomas are primarily resistant to conventional radiation and chemotherapy [1,3,4]. Surgical resection remains the primary treatment option [5]. For patients with unresectable disease, the prognosis is dismal and symptoms can be debilitating due to sites of disease involvement.

Recent advances in the molecular understanding of sarcomas and the development of targeted therapy for sarcoma treatment have led to interest in the possibility of testing targeted agents in chondrosarcomas [6]. Gene expression profiling has identified high levels of tyrosine kinase and receptor tyrosine kinase expression in a number of sarcoma types, indicating that sarcomas may potentially be candidates for therapy with tyrosine kinase inhibitors [7-14]. In chondrosarcoma, the platelet derived growth factor receptor (PDGFR) tyrosine kinase pathway has also been shown to be activated, as evidenced by the overexpression of both PDGFR-α and -β and increased PDGFR signaling activity [15,16]. Due to its inhibition of the PDGFR tyrosine kinase pathway, we hypothesize that sunitinib would have beneficial activity in chondrosarcoma.

## Case presentation

**Figure 1:**
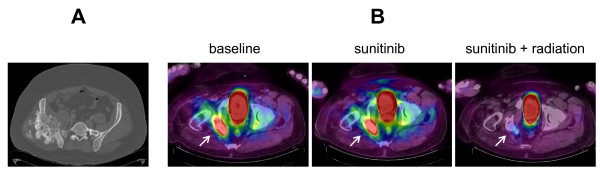
**Representative scans done upon diagnosis and during the course of sunitinib treatment**. **(A) **Diagnostic computed tomography showing the multilobulated chondrosarcoma mass arising from his right pelvis. **(B) **Superimposed fluorine-18- fluorodeoxyglucose-positron emission tomography and computed tomography scans showing the pelvis mass (white arrow) immediately prior to sunitinib, two months after initiation of sunitinib alone and two months after combined sunitinib and proton beam radiation. Intensity of fluorine-18-fluorodeoxyglucose uptake: red, high; yellow, intermediate; blue, low.

**Figure 2:**
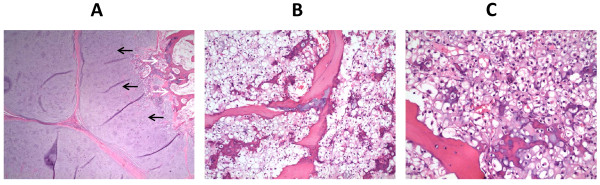
**Hematoxylin and eosin staining of sections from our patient's pelvic tumor mass and skull metastasis**. **(A) **Low-power photomicrograph demonstrating the low-grade chondrosarcoma (black arrows) arising in the stalk of a pre-existing osteochondroma (white arrows). **(B) **Focally within the stalk of the osteochondroma, the tumor had features of clear cell chondrosarcoma. **(C) **The skull metastasis consisted entirely of the clear cell chondrosarcoma component.

We present the case of a 32-year-old Caucasian man who initially presented five years previously with right hip pain and was found at that time to have a large multilobulated mass arising from his right ilium and extending to his right sacrum with involvement of his gluteal musculature (Figure 1A). A biopsy showed a grade 1 chondrosarcoma. Subsequently, a right hemipelvectomy was performed, and the diagnosis of grade 1 chondrosarcoma arising from a pre-existing osteochondroma was confirmed (Figure 2A). Additionally, a minor component of clear cell chondrosarcoma was identified at the origin of the osteochondroma within the medullary cavity of the underlying ileum (Figure 2B). Recurrence in the sacral stump and an area adjacent to his paraspinous muscles was noted two years later after our patient experienced a fall. He underwent proton beam radiotherapy and resection, complicated by a postsurgical abscess.

Approximately two-and-a-half years later, our patient presented with significant pain in his right pelvis, difficulty in ambulating and a decreased performance status due to pain. A bone scan showed uptake in his left temporal bone and an excisional biopsy confirmed recurrence of the clear cell chondrosarcoma (Figure 2C). A positron emission tomography/computed tomography (PET/CT) scan showed uptake to an standardized uptake value (SUV) maximum of 9.0 in the right sacral lesion with two other areas of uptake in his pelvis (Figure 1B). Two non-hypermetabolic pulmonary nodules were also noted. Stereotactic radiosurgery was applied to the temporal bone lesion. Our patient was started on sunitinib 37.5 mg daily, which was initially tolerated well, with only mild fatigue. His pain greatly improved. A PET/CT scan obtained two months after the initiation of sunitinib showed improvement in the fluorine-18-fluorodeoxyglucose (FDG)-avidity of the lesions, with a right sacral lesion showing an SUV of 6.8 (Figure 1B). He continued to take sunitinib at the same dose and was referred for proton beam radiation to his right pelvis. He received twenty fractions of radiation concurrent with sunitinib 37.5 mg daily and experienced some diarrhea and worsening of an acneiform rash, but our patient wished to continue on the same dose given his excellent response with regards to the pain. A total of six months after initiating sunitinib, our patient continued to show improvement on imaging, with a PET/CT scan revealing stable non-hypermetabolic pulmonary nodules and improvement in the right sacral lesion with an SUV of 3.4 (Figure 1B). He did require a reduction in the dose 25 mg daily, however, due to the worsening acneiform rash and diarrhea.

## Discussion

Sunitinib is a multitargeted receptor tyrosine kinase inhibitor that exhibits inhibitory activity against multiple targets including c-KIT, vascular endothelial growth factor receptors (VEGFR; VEGFR1, VEGFR2 and VEGFR3), PDGFR-α and -β, fms-like tyrosine kinase receptor-3, colony stimulating factor 1 receptor, and RET [17,18]. Inhibition of these receptor tyrosine kinases blocks the transduction of signals important for tumor growth, survival and angiogenesis. Phase II and III clinical trials with sunitinib have shown clinical benefit, with significantly improved progression-free and overall survival in patients with imatinib-resistant gastrointestinal stromal tumors (GIST) and advanced renal cell carcinoma [19,20]. A phase II study showed that continuous use of sunitinib in advanced or metastatic non-GIST sarcomas produced a noteworthy metabolic response in many patients [11].

In a murine model, sunitinib was shown to be synergistic with radiation in increasing apoptosis in endothelial cells, enhancing destruction of the tumor vasculature and increasing time to tumor growth [21]. While tumors regrew rapidly after the discontinuation of sunitinib in this study, it was also shown that restarting sunitinib therapy resulted in further growth delays, suggesting that patients can benefit from maintenance therapy with sunitinib after the completion of radiation.

Kao *et al*. published results of a phase I study employing sunitinib concurrent with stereotactic radiation for patients with oligometastatic disease [22]. In the study, 21 patients with one to five sites of metastatic disease of any solid tumor type were treated with sunitinib, starting at 25 mg daily, with radiation to 40Gy in 10 fractions. The doses of both the radiation and the sunitinib were increased as tolerated, leading to a dosing recommendation for sunitinib with radiation at 37.5 mg daily. Sunitinib in conjunction with radiation yielded a complete response in 42% of the lesions, with a partial response in 17% and stable disease in 28%. At the last follow-up, 38% of these patients were without evidence of disease.

## Conclusions

Our study is the first to show that sunitinib has beneficial activity in chondrosarcoma and may be safely combined with proton beam radiation therapy. Randomized phase II or III clinical trials are needed to assess the role of sunitinib in this disease and as a radiosensitizer.

## Consent

Written informed consent was obtained from the patient for publication of this case report and any accompanying images. A copy of the written consent is available for review by the Editor-in-Chief of this journal.

## Competing interests

The authors declare that they have no competing interests.

## Authors' contributions

All authors contributed to the care of the patient and analyzed the data. JDR performed the histological examination of the tumor sections. JD and LHD drafted the manuscript. All authors read and approved the final manuscript.
